# The association between nicotine dependence and sleep quality in patients referred to a smoking cessation outpatient clinic: A cross-sectional study

**DOI:** 10.18332/tid/194170

**Published:** 2024-11-04

**Authors:** Umran Ozden Sertcelik, Aysegul Karalezli

**Affiliations:** 1 Department of Chest Diseases, Faculty of Medicine, Ankara Yıldırım Beyazıt University, Ankara, Türkiye; 2 Department of Chest Diseases, Ankara Bilkent City Hospital, Ankara, Türkiye

**Keywords:** anxiety disorder, sleep hygiene, sleepiness, tobacco use disorder, tobacco use cessation

## Abstract

**INTRODUCTION:**

Nicotine addiction is one of the most common forms of dependence. It is shown to be associated with many chronic diseases that develop mostly through smoking. The association between sleep quality and smoking or nicotine addiction has not been clarified yet. This study aimed to investigate the association between nicotine addiction and sleep quality.

**METHODS:**

In this cross-sectional study, the Epworth sleepiness scale (ESS), Pittsburgh sleep quality index (PSQI), Fagerström test for nicotine dependence (FTND), and Hospital anxiety-depression scale (HADS) were administered to patients who applied to the smoking cessation outpatient clinic of a reference hospital between April and June 2023. FTND measured nicotine dependence, and its association with sleep quality estimated by PSQI was assessed by binary logistic regression with the potential confounders.

**RESULTS:**

In the study group of 280 participants, 67.1% were male, and 57.8% had poor sleep quality. The median (IQR) FNTD score was 7.0 (3.0), and the median global PSQI score was 6.0 (5.0). Poor sleep quality increased by 1.12 times (AOR=1.12; 95% CI: 1.02–1.22, p=0.016) for each unit increase in hospital anxiety score and by 1.22 times (AOR=1.22; 95% CI: 1.05–1.42, p=0.011) for each unit increase in FTND score.

**CONCLUSIONS:**

Increased nicotine dependence and anxiety are independently associated with poor sleep quality. The findings support smoking cessation efforts. It is recommended to study the effect of combating anxiety and tobacco addiction to improve sleep quality.

## INTRODUCTION

Nicotine dependence is one of the most common forms of dependence in Türkiye and in the world^1,2^. Nicotine dependence develops with tobacco products, especially cigarettes in Türkiye, with a smoking prevalence of >30%, which also makes Türkiye one of the most prevalent countries^2^.

Besides the high prevalence, the World Health Organization reports that more than 7 million people die every year due to smoking. It contributes to the increasing burden of morbidity. Although its association with many chronic diseases is strongly demonstrated^3^, its association with depression, anxiety, and sleep quality has not yet been demonstrated.

Low sleep quality is associated with many mental illnesses and causes negative consequences such as occupational accidents and traffic accidents. Quality sleep is important for physical, mental, and social health^4^. So, it is valuable to identify the causes of poor sleep quality and to reduce modifiable risks.

It is thought that sleep apnea may develop due to changes in the amount of neurotransmitters released from the central nervous system with long-term exposure to the chemicals in the structure of cigarettes and inflammatory processes in the upper airways and that hypercarbia, hypoventilation, and central apnea conditions due to smoking may affect sleep quality^4,5^.

Based on this idea, it is believed that there may be an association between sleep quality and nicotine dependence/smoking behavior. Research on the subject is limited in the literature. Determining this association epidemiologically will create another motivation to combat nicotine dependence. As the evidence on the subject increases, it can be used as an output in evaluating the results of the fight against nicotine dependence.

This study aimed to examine the sleep quality status and the association between nicotine dependence level and sleep quality among patients who applied to a smoking cessation outpatient clinic.

## METHODS

This cross-sectional study was conducted on patients who applied to Ankara Bilkent City Hospital Smoking Cessation Outpatient Clinic of Chest Diseases between April and July 2023 in Ankara, Türkiye. The study center is a reference center with 4200 beds capacity and an intensive care unit of 700 beds capacity^6^.

Patients who gave informed consent and were aged >18 years were included in the study. Pregnant and breastfeeding women, night-shift workers, patients with any addiction other than smoking, patients with any psychiatric disease other than a history of anxiety and depression, patients with a diagnosis of obstructive sleep apnea syndrome (OSAS) and body mass index ≥30 kg/m^2^ were excluded. All eligible individuals were included in the study, but no sample was selected. Patients with alcohol and substance addiction are referred to psychiatry before applying to the smoking cessation outpatient clinic. There were no patients with alcohol and substance addiction in the study group.

A standardized data collection form created for the study was used to record age, gender, height (m), weight (kg), chronic diseases, age at initiation of smoking, duration, and the number of cigarettes smoked per day. Participants completed the Epworth sleepiness scale (ESS), Pittsburgh Sleep Quality Index (PSQI), Fagerström test for nicotine dependence (FTND), and Hospital anxiety-depression scale (HADS) under supervision. Sleep quality was assessed based on the global PSQI score. Those with a score of ≤5 were categorized as having good sleep quality, and those >5 were categorized as poor sleep quality, as suggested in the original study^7^.

### Fagerström test for nicotine dependence

This involves a questionnaire consisting of six questions to assess dependence related to cigarette smoking. It consists of ‘yes/no’ questions scored as 0 or 1, and multiple-choice questions that can be scored 0–3. Dependence is evaluated with a score 0–10 points from the lowest to the highest^8^ according to: 1–2 low, 3–4 low-moderate, 5–7 moderate, and ≥8 high. It is routinely administered to all patients presenting to outpatient clinics for tobacco dependence. Uysal et al.^9^ conducted a Turkish validity and reliability study (Cronbach’s alpha 0.56).

### Pittsburgh Sleep Quality Index (PSQI)

It is a subjective sleep quality index in which the sleep quality of the patients in the last month is evaluated by themselves. The individual can answer 19 questions, and the spouse or roommate can answer 5 questions. If there is no spouse or roommate, the calculation is based on 19 questions. In this study, 19 questions were evaluated. Seven component scores are calculated for these questions: subjective sleep quality, sleep latency, sleep duration, habitual sleep efficiency, sleep disturbances, use of sleeping medication, and daytime dysfunction. The sum of the scores (0–3) of these seven components gives a global score. The global score can range from 0 to 21, increasing from good to poor sleep quality. Scores of ≤5 are categorized as good sleep quality, and scores >5 as poor sleep quality. In the original validity and reliability study, sensitivity was reported as 89.6%, specificity as 86.5%, and Cronbach’s alpha was 0.83^7^. In the Turkish validity and reliability study, Cronbach’s alpha was 0.80^10^.

### Epworth sleepiness scale (ESS)

The ESS is a self-administered questionnaire. It consists of eight questions that subjectively assess daytime sleepiness; each question can be scored between 0 and 3. In total, a score between 0 and 24 is obtained. Scores of ≤10 indicate normal daytime sleepiness, 11–14 mild, 15–17 moderate, and ≥18 severe. Cronbach’s alpha was 0.88 for the internal validity of the original scale^11^ and Cronbach’s alpha was 0.86 for the internal validity of the Turkish form^12^.

### Hospital anxiety-depression scale (HADS)

Outpatients admitted to the hospital evaluate 7 items related to depression and 7 items related to anxiety by marking one of the four options (corresponding to 0–3 points) that are most appropriate for them, taking into account the last few days. A total of 0–21 points can be obtained separately for depression and anxiety. Scores of ≤7 are considered normal, 8–10 mild, 11–14 moderate, and 15–21 severe. The validity and reliability study was conducted by Zigmond et al.^13^. The Turkish validity and reliability study was conducted by Aydemir et al.^14^ Cronbach’s alpha was 0.85 for the anxiety subscale and 0.78 for the depression subscale.

### Statistical analysis

Descriptive statistics were reported using mean and standard deviation (SD) or median and interquartile range (IQR) for continuous variables, and frequencies and percentages for categorical variables. Pairwise comparisons were performed using Pearson’s chi-squared test for categorical variables and Student’s t-test or Mann-Whitney U test for continuous variables. Binary logistic regression was used to determine the association between sleep quality and nicotine dependence. Age, gender, underlying disease, depression, and anxiety were considered as possible confounders. Nicotine dependence was included in the model as the exposure, age at initiation of smoking, and cigarettes per day were included as covariates. Adjusted odds ratios (AOR) and 95% confidence intervals are reported. The goodness of fit was tested with the Hosmer-Lemeshow test. A p<0.05 (two-sided) was considered statistically significant. Statistical analysis was carried out using the Statistical Package for the Social Sciences (SPSS) version 23.0 (IBM Corp, Armonk, NY, USA).

Retrospective power analysis was performed with the *epiR* package version 2.0.67 in R program version 4.2.2. The power was 99.8%. Violin plots were produced with the *ggplot2* package version 3.4.3 in R program version 4.2.2.

### Ethical considerations

The study protocol was ethically approved by the Ankara Bilkent City Hospital Clinical Research Ethics Committee No. 1 (Approval number: E1-23-3554, date: May 10, 2023). This study was performed in line with the principles of the Declaration of Helsinki. Patient written informed consent was obtained.

## RESULTS

**Figure 1:**
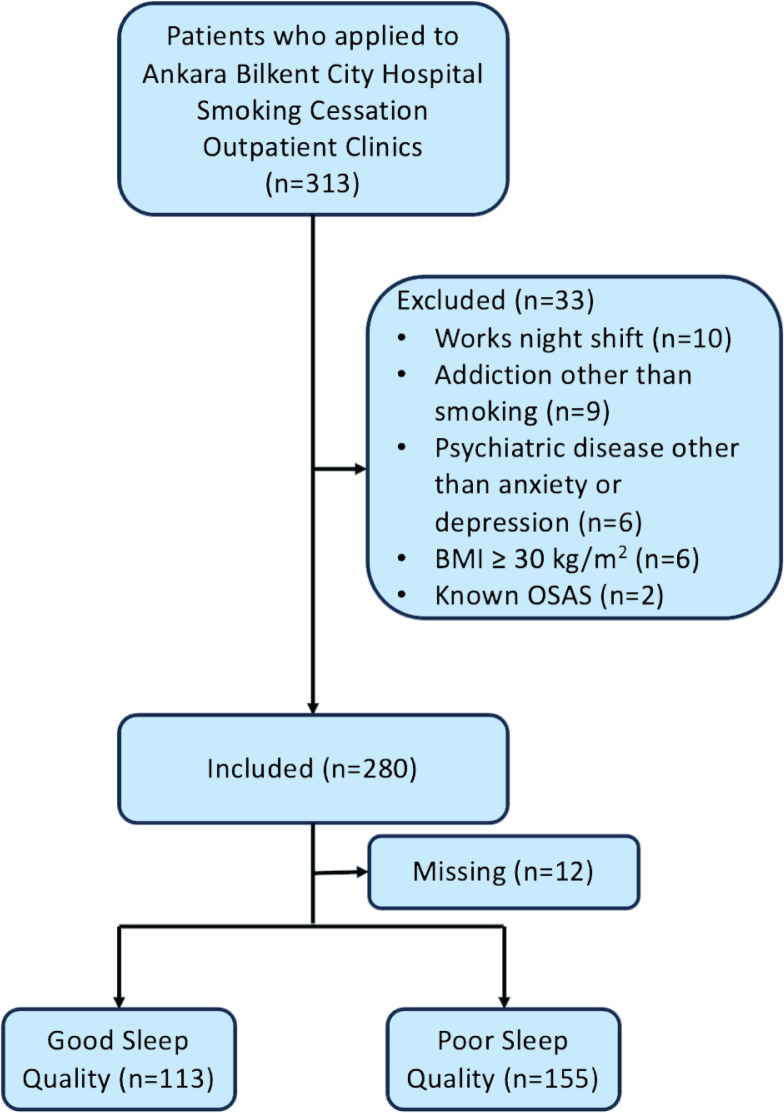
Flow chart of selection of patients who applied to the smoking cessation outpatient clinic of Ankara Bilkent City Hospital, April – June 2023, Türkiye (N=280)

Of the 313 patients who met the inclusion criteria, 10 were excluded because they worked night shifts, 9 had an addiction other than smoking, 6 had a psychiatric disease other than anxiety or depression, 6 had a BMI of ≥30 kg/m^2^, and 2 had a known diagnosis of OSAS (Figure 1). There were 280 patients in the study group, 67.1% (n=188) were male, and the mean age was 38.0 ± 12.0 years. The mean global PSQI score was 6.63 ± 3.09 and 113 (42.2%) of the participants had good sleep quality. The distribution of scores for the seven components of the PSQI is given in Table 1.

**Table 1:** Distribution in Pittsburgh Sleep Quality Index components of patients who applied to the smoking cessation outpatient clinic of Ankara Bilkent City Hospital, April – June 2023, Türkiye (N=280)

*Characteristics*	*n*	*%*
**Component 1: Subjective sleep quality** (Q9)		
Very good (0 points)	34	12.7
Fairly good (1 point)	121	45.1
Fairly bad (2 points)	94	34.7
Very bad (3 points)	19	6.9
**Component 2: Sleep latency** (Q2 + Q5a)		
0 (0 points)	56	20.9
1–2 (1 point)	81	30.2
3–4 (2 points)	94	35.1
5–6 (3 points)	37	13.8
**Component 3: Sleep duration** (hours) (Q4)		
>7 (0 points)	117	43.7
6–7 (1 point)	61	22.8
5–6 (2 points)	57	21.3
<5 (3 points)	33	12.3
**Component 4: Sleep efficiency** (%) (Q1, Q3, Q4)		
>85 (0 points)	240	89.6
75–84 (1 point)	11	4.1
65–74 (2 points)	10	3.7
<65 (3 points)	7	2.6
**Component 5: Sleep disturbances** (Q5 b–j)		
0 (0 points)	3	1.1
1 – 9 (1 point)	144	53.7
10 – 18 (2 points)	100	37.3
19 – 27 (3 points)	21	7.8
**Component 6: Sleep medication** (Q6)		
Not during the past month (0 points)	245	91.4
<1 per week (1 point)	4	1.5
1–2 per week (2 points)	5	1.9
≥3 per week (3 points)	14	5.2
**Component 7: Daytime dysfunction** (Q7 + Q8)		
0 (0 points)	111	41.4
1–2 (1 point)	86	32.1
3–4 (2 points)	56	20.9
5–6 (3 points)	15	5.6
**PSQI score** (N=268)		
Good sleep quality (≤5 points)	113	42.2
Poor sleep quality (>5 points)	155	57.8
Global, mean ± SD, median (IQR)	6.63 ± 3.09	6.00 (5.00)

PSQI: Pittsburgh Sleep Quality Index. IQR: interquartile range. SD: standard deviation.

The mean BMI of the participants was 25.4 ± 4.7 kg/m^2^. The mean age at initiation of smoking was 16.8 ± 4.4 years, the mean duration of smoking was 20.4 ± 11.5 years and the mean number of cigarettes smoked per day was 24.9 ± 10.9. The median (IQR) FNTD score was 7.0 (3.0), the HADS anxiety score was 8.0 (6.0), the HADS depression score was 6.0 (6.0), the ESS score was 6.0 (6.0) and global PSQI score was 6.0 (5.0). The patient demographic and smoking characteristics, and their scores for nicotine dependence, hospital anxiety-depression, ESS, and PSQI are presented in Table 2.

**Table 2:** Demographic, smoking and hospital anxiety-depression characteristics of patients who applied to the smoking cessation outpatient clinic of Ankara Bilkent City Hospital, April – June 2023, Türkiye (N=280)

*Characteristics*	*n*	*Mean ± SD*	*Median (IQR)*
**Demographic**			
Age (years)	280	38.0 ± 12.0	37.0 (18.0)
Body mass index (kg/m^2^)	279	25.4 ± 4.7	25.3 (6.1)
**Smoking**			
Starting age of smoking (years)	280	16.8 ± 4.4	16.0 (4.0)
Length of smoking (years)	280	20.4 ± 11.5	19.0 (17.0)
Cigarettes per day	275	24.9 ± 10.9	20.0 (10.0)
FTND score	280	6.5 ± 2.3	7.0 (3.0)
**Hospital anxiety-depression**			
Anxiety score	279	8.2 ± 4.4	8.0 (6.0)
Depression score	279	6.5 ± 4.0	6.0 (6.0)
Global PSQI score	268	6.6 ± 3.1	6.0 (5.0)
Epworth sleepiness score	280	6.5 ± 4.4	6.0 (6.0)

FTND: Fagerström test for nicotine dependence. PSQI: Pittsburgh sleep quality index. IQR: interquartile range. SD: standard deviation.

**Figure 2:**
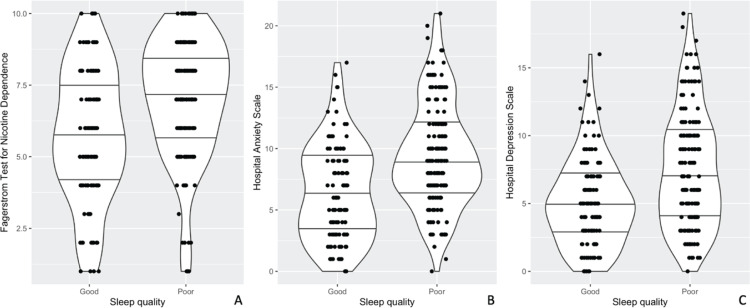
Violin plots of Fagerström test for nicotine dependence (A), hospital anxiety (B), and depression (C) scale scores by sleep quality of patients who applied to the smoking cessation outpatient clinic of Ankara Bilkent City Hospital, April – June 2023, Türkiye (N=280)

The distribution of demographic, comorbidity, smoking status, nicotine dependence, hospital anxiety-depression, and daytime sleepiness status of the participants according to poor and good sleep quality is shown in Table 3. The median age was 38.0 (21.0) years in the group with good sleep quality and 36.0 (18.0) years in the group with poor sleep quality, and a statistically significant difference was found between the groups (p=0.032). The history of depression was 2.19 (95% CI: 1.07–4.46; p=0.029) times higher in the group with poor sleep quality than in the group with good sleep quality. Age at smoking initiation was lower in the group with poor sleep quality than in the group with good sleep quality (p=0.048). The number of cigarettes per day was higher in patients with poor sleep quality than in patients with good sleep quality (p=0.026). FNTD score was higher in the group with poor sleep quality than in the group with good sleep quality when the lower group was taken as a reference (p<0.001). HADS and ESS were higher in the group with poor sleep quality (all p<0.001) (Figure 2).

**Table 3:** Demographic, disease, smoking, and hospital anxiety-depression characteristics by sleep quality of patients who applied to the smoking cessation outpatient clinic of Ankara Bilkent City Hospital, April – June 2023, Türkiye (N=280)

*Characteristics*	*Sleep quality*			*p*
	*Poor*	*Good*	*OR (95% CI)*	
	*n (%)*	*n (%)*		
**Demographic**				
Age (years), median (IQR)	36.0 (18.0)	38.0 (21.0)	-	**0.032** ^a^
Males	103 (66.5)	78 (69.0)	0.89 (0.53–1.50)	0.657^b^
**Disease**				
Underlying diseases	66 (42.6)	36 (31.9)	1.59 (0.95–2.64)	0.074^b^
Depression	32 (20.6)	12 (10.6)	2.19 (1.07–4.46)	**0.029** ^b^
Hypertension	22 (14.2)	9 (8.0)	1.91 (0.85–4.33)	0.115^b^
Asthma	10 (6.5)	7 (6.2)	1.04 (0.39–2.83)	0.932^b^
Coronary artery disease	10 (6.5)	6 (5.3)	1.23 (0.43–3.48)	0.697^b^
Chronic obstructive pulmonary disease	8 (5.2)	6 (5.3)	0.97 (0.33–2.88)	0.957^b^
Diabetes mellitus	8 (5.2)	4 (3.5)	1.48 (0.44–5.05)	0.526^b^
**Body mass index** (kg/m^2^), median (IQR)	25.1 (5.8)	25.5 (6.3)	-	0.222^a^
Underweight (<18.5)	8 (5.2)	5 (4.4)	1.02 (0.31–3.63)	0.265^b^
Healthy weight (18.5–24.9)	69 (44.5)	44 (38.9)	1.00	
Overweight (25.0–29.9)	59 (38.1)	40 (35.4)	0.94 (0.54–1.63)	
Obese (≥30.0)	19 (12.3)	24 (21.2)	0.51 (0.25–1.03)	
**Smoking**				
Starting age of smoking (years), median (IQR)	16.0 (4.0)	17.0 (4.0)	-	**0.048** ^a^
Length of smoking (years), median (IQR)	17.0 (17.0)	20.0 (17.0)	-	0.204^a^
Cigarettes per day, median (IQR)	23.0 (10.0)	20.0 (10.0)	-	**0.026** ^a^
**FTND score,** median (IQR)	7.0 (3.0)	6.0 (4.0)	-	**<0.001^a^**
Low (1–2 points)	8 (5.2)	13 (11.5)	1.00	**<0.001** ^b^
Low-moderate (3–4 points)	7 (4.5)	20 (17.7)	0.57 (0.17–1.95)	
Moderate (5–7 points)	67 (43.2)	49 (43.4)	2.22 (0.86–5.77)	
High (≥8 points)	73 (47.1)	31 (27.4)	3.83 (1.44–10.15)	
**Hospital anxiety-depression**				
**Anxiety score,** median (IQR)	9.0 (5.0)	6.0 (6.0)		**<0.001** ^a^
Normal (<8 points)	57 (36.8)	66 (58.9)	1.00	**<0.001** ^b^
Mild (8–10 points)	45 (29.0)	30 (26.8)	1.73 (0.97–3.11)	
Moderate (11–14 points)	29 (18.7)	12 (10.7)	2.80 (1.31–5.99)	
Severe (15–21 points)	24 (15.5)	4 (3.6)	6.95 (2.28–21.21)	
**Depression score,** median (IQR)	7.0 (6.0)	5.0 (4.0)	-	**<0.001** ^a^
Normal (<8 points)	84 (54.2)	88 (78.6)	1.00	**<0.001** ^b^
Mild (8–10 points)	36 (23.2)	16 (14.3)	2.36 (1.22–4.56)	
Moderate (11–14 points)	25 (16.1)	7 (6.3)	3.74 (1.54–9.11)	
Severe (15–21 points)	10 (6.5)	1 (0.9)	10.48 (1.31–83.62)	
**Epworth sleepiness score,** median (IQR)	7.0 (6.0)	4.0 (6.0)		**<0.001** ^a^
Normal (0–10 points)	127 (81.9)	101 (89.4)	1.00	0.091^b^
Abnormal (>10 points)	28 (18.1)	12 (10.6)	1.86 (0.90–3.83)	

FTND: Fagerström test for nicotine dependence. IQR: Interquartile range.

a Mann-Whitney U test p-value.

b Pearson’s chi-squared test or Fisher’s exact test p-value.

Factors associated with poor sleep quality were analyzed by binary logistic regression analysis and are presented in Table 4. Age, male gender, presence of comorbidity, age at initiation of smoking, cigarettes per day, and HADS and FTND scores were included in the model. Each 1-point increase in FNTD score increased having poor sleep quality 1.22 times (AOR=1.22; 95% CI: 1.05–1.42; p=0.011), and each 1-point increase in hospital anxiety score increased having poor sleep quality 1.12 times (AOR=1.12; 95% CI: 1.02–1.22; p=0.016).

**Table 4:** Logistic regression analysis of factors associated with poor sleep quality of patients who applied to the smoking cessation outpatient clinic of Ankara Bilkent City Hospital, April – June 2023, Türkiye (N=280)

*Variables*	*Univariate*			*Multivariate*		
	*OR*	*95% CI*	*p*	*AOR*	*95% CI*	*p*
Age (years)	0.98	0.96–1.00	**0.028**	0.98	0.96–1.01	0.147
Male gender	0.89	0.53–1.50	0.657	0.86	0.46–1.61	0.859
Underlying disease	1.59	0.95–2.64	0.075	1.62	0.90–2.90	0.107
Starting age of smoking (years)	0.93	0.87–0.98	**0.013**	0.97	0.91–1.04	0.394
Cigarettes per day	1.03	1.00–1.05	**0.031**	1.00	0.97–1.04	0.829
Hospital anxiety score	1.20	1.12–1.29	**<0.001**	1.12	1.02–1.22	**0.016**
Hospital depression score	1.17	1.09–1.26	**<0.001**	1.06	0.97–1.16	0.221
FTND score	1.30	1.15–1.46	**<0.001**	1.22	1.05–1.42	**0.011**

The model was completed with 263 participants. Nagelkerke’s R^2^=0.235, Hosmer-Lemeshow test p=0.201. AOR: adjusted odds ratio. FTND: Fagerström test for nicotine dependence.

## DISCUSSION

The association between nicotine dependence and sleep quality was examined in patients who applied to the adult smoking cessation outpatient clinic of a reference center. It was found that each point increase in the Fagerström nicotine dependence score increased poor sleep quality by 1.22 times. This association was also revealed when confounding conditions, especially anxiety and depression, were controlled for. Having high nicotine dependence is associated with poor sleep quality.

A study had been conducted with 350 participants, a total of 88 smokers, consisting of residents and interns working at Lebanon University Faculty of Medicine. It was determined that 67 (76.1%) of the smokers and 21 (9.1%) of the non-smokers had poor sleep quality (p<0.001), and it was shown that having a high nicotine dependence score was associated with poor sleep quality (OR=4.69; 95% CI: 2.17–10.10, p<0.001), similar to the results of this study^15^. However, the association was higher than in this study because this group also included non-smokers. In the meta-analysis investigating the effect of smoking on sleep quality in Türkiye, data from a total of 6698 participants were evaluated; 66.2% of the participants were male, and 57% had poor sleep quality. According to the meta-analysis results, the sleep quality in non-smokers is 1.7 times better than in smokers (OR=1.7; 95% CI: 1.50–1.92; p<0.0001)^16^.

These findings strengthen the idea that smoking and poor sleep quality occur through nicotine addiction. The present study was conducted only in smokers. However, nicotine dependence maintains its significance in the model, including the age of starting smoking and the average cigarettes per day, so both the duration and quantity of smoking are taken into account. A study conducted in Lebanon supported the findings of this study. The fact that the frequency of cocaine use is associated with poor sleep quality provides an analogy in terms of addiction. On the other hand, it is claimed that nicotine addiction may disrupt the normal sleep cycle by altering the levels of neurotransmitters such as acetylcholine, dopamine, and serotonin, which are responsible for regulating sleep and wakefulness in the brain^17^. This supports our study findings in terms of biological mechanisms.

In terms of anxiety, as a possible confounding effect, a high anxiety score was found to be associated with poor sleep quality. The fact that addiction and anxiety are independently associated with poor sleep quality is stimulating for people with these two conditions together. The results of the state-trait anxiety inventory and FNTD applied to 503 university students during the pandemic period in Türkiye were compared. A statistically positive and significant correlation was found between nicotine dependence scores and mean scores of state-trait anxiety inventory (r=0.106, p=0.017)^18^. In a study conducted in Israel, PSQI, FNTD, Beck depression inventory, and state-trait anxiety inventory were applied to smokers and non-smokers. No association was found between anxiety and depression scores and sleep efficiency and subjective sleep quality^3^. Here, it is thought that the number of participants may not be sufficient to show the association. In a study investigating the association between anxiety and sleep quality in 46387 adolescents in Korea in 2020, it was found that anxiety was higher in participants with poor sleep quality in both genders (AOR=1.56; 95% CI: 1.43–1.71 in males; AOR=1.30; 95% CI: 1.19–1.42 in females)^19^.

### Limitations

Within this study, the association controlled for confounders including anxiety and depression. However, this study was conducted in a single center, and the generalizability of the results is limited. Since it is based on self-report, it has the potential to be affected by recall bias. It also may be influenced by social-desirability bias. However, it may have a limited effect and may be biased towards the null since the participants were applied to the smoking cessation outpatient clinic. Due to its cross-sectional design, temporality is unknown, and a causal relationship cannot be claimed.

## CONCLUSIONS

There is an association between high nicotine addiction and poor sleep quality among people applying to the smoking cessation clinic. Since an independent association has been found between high anxiety and poor sleep quality, it is considered that people with both conditions are more prone to poor sleep quality and the negative consequences associated with poor sleep quality. It is recommended to examine the effects of interventions to quit smoking and cope with anxiety on sleep quality.

## Data Availability

The data supporting this research are available from the authors on reasonable request. Data are not publicly available.
